# Public Reactions to the Cigarette Control Regulation on a Chinese Microblogging Platform: Empirical Analysis

**DOI:** 10.2196/14660

**Published:** 2020-04-27

**Authors:** Wanting Wen, Zhu Zhang, Ziqiang Li, Jiaqi Liang, Yongcheng Zhan, Daniel D Zeng, Scott J Leischow

**Affiliations:** 1 State Key Laboratory of Management and Control for Complex Systems Institute of Automation Chinese Academy of Sciences Beijing China; 2 School of Artificial Intelligence University of Chinese Academy of Sciences Beijing China; 3 Shenzhen Artificial Intelligence and Data Science Institute (Longhua) Shenzhen China; 4 Orfalea College of Business California Polytechnic State University San Luis Obispo, CA United States; 5 College of Health Solutions Arizona State University Phoenix, AZ United States

**Keywords:** cigarette smoking, regulations, social media, information networks

## Abstract

**Background:**

On January 1, 2019, a new regulation on the control of smoking in public places was officially implemented in Hangzhou, China. On the day of the implementation, a large number of Chinese media reported the contents of the regulation on the microblog platform Weibo, causing a strong response from and heated discussion among netizens.

**Objective:**

This study aimed to conduct a content and network analysis to examine topics and patterns in the social media response to the new regulation.

**Methods:**

We analyzed all microblogs on Weibo that mentioned and explained the regulation in the first 8 days following the implementation. We conducted a content analysis on these microblogs and used social network visualization and descriptive statistics to identify key users and key microblogs.

**Results:**

Of 7924 microblogs, 12.85% (1018/7924) were in support of the smoking control regulation, 84.12% (6666/7924) were neutral, and 1.31% (104/7924) were opposed to the smoking regulation control. For the negative posts, the public had doubts about the intentions of the policy, its implementation, and the regulations on electronic cigarettes. In addition, 1.72% (136/7924) were irrelevant to the smoking regulation control. Among the 1043 users who explicitly expressed their positive or negative attitude toward the policy, a large proportion of users showed supportive attitudes (956/1043, 91.66%). A total of 5 topics and 11 subtopics were identified.

**Conclusions:**

This study used a content and network analysis to examine topics and patterns in the social media response to the new smoking regulation. We found that the number of posts with a positive attitude toward the regulation was considerably higher than that of the posts with a negative attitude toward the regulation. Our findings may assist public health policy makers to better understand the policy’s intentions, scope, and potential effects on public interest and support evidence-based public health regulations in the future.

## Introduction

### Background

China has been the largest consumer market of cigarettes in the world. In China, approximately 27.7% of adults aged between 15 and 69 years and 1% of adolescents aged between 13 and 15 years are smokers [1]. According to a World Health Organization (WHO) report, more than 1 million people die from smoking-related diseases every year in China, whereas 740 million people including 180 million children suffer from secondhand smoking [2]. In addition to the high rates of cigarette use, electronic cigarettes (e-cigarettes) have become increasingly popular in China, especially among adolescents [3]. E-cigarette sellers claim that e-cigarettes are less harmful than tobacco and help to quit smoking [4]. Some research shows that e-cigarettes appear to be effective when used by smokers as an aid to quit smoking, and the hazard to health arising from long-term vapor inhalation from the e-cigarettes available is likely to be much less than the harm from smoking tobacco [5,6]. However, some research also shows the risks associated with e-cigarette use. The inhaled aerosols of e-cigarettes contain numerous potential toxins, some of which could be dangerous for health with long-term use [7], and inhalation of the non-nicotine–derived chemicals present in e-cigarette aerosols is actually harmful to small airway epithelium cells and alveolar macrophages in e-cigarette smokers. As the respiratory system of minors is not fully developed, inhalation of such aerosols can have adverse effects on lung function [8-10]. Improper use may also lead to various safety risks such as nicotine poisoning, and it is possible that youth using a new generation of e-cigarettes will become addicted [9]. Widespread smoking and tobacco use and the uncertainty of e-cigarette effects, including the potential of youth addiction to both cigarettes and e-cigarettes, raise severe public health concerns.

To alleviate the above challenges, the government agencies in China have taken actions to increase tobacco control regulations. In 2005, China ratified the WHO’s Framework Convention on Tobacco Control to reduce the harms from smoking [11]. On November 24, 2014, the Health and Family Planning Commission of China drafted the “Regulations on the Control of Smoking in Public Places (Draft for Review)” [12] to solicit opinions from the public. This is the first time that China has proposed to formulate administrative regulations to comprehensively control tobacco throughout the country [12]. According to the statistics of the Health and Family Planning Commission of China, until the end of 2016, 18 cities in China had formulated local smoke-free environmental laws and regulations, covering one-tenth of the total population of China [13]. In recent years, tobacco control efforts in China have been strengthened and tobacco taxes have increased. As a result, smokers began to choose e-cigarettes as a nicotine delivery tool [14]. With the prominent usage of e-cigarettes among teenagers and more studies being conducted on the harm of e-cigarettes on minors, China issued the “Notice on Prohibiting the Sale of Electronic Cigarettes to Minors” in 2018 [10], which was the first of several regulations on e-cigarette control in China, to protect minors from e-cigarettes.

Social media platforms play an important role in spreading news and shaping the public’s attitudes toward novel health issues [15,16]. Different groups of users can use different social media platforms to discuss smoking topics [17]. Public health community and policy makers can use these platforms for public health surveillance [18-20]. For example, for a newly issued policy, public health officials can use these platforms to judge the public's understanding of the policy to determine whether the policy needs to be further interpreted and publicized. By exploring the user-generated content on social media, researchers can study public opinions on cigarette use [21] and understand the influence of tobacco control policies on the smoking cessation intentions [22,23]. There have been some studies about public health on social media in the United States [17-20]. However, related research is sparse in China. Only a few research studies have focused on social media studies regarding the public health strategies about cigarette and e-cigarette [24-26]. For example, Cui et al [25] explored the nature and extent of discussions around the electronic nicotine delivery systems in Chinese social media, which have the power to influence a massive audience. Jin et al [26] evaluated the effectiveness of microblogging in tobacco control communication and provided new media era strategies for tobacco control. Thus, there is an urgent need to conduct social media–driven public opinion studies on smoking control policies.

### Objectives

On January 1, 2019, the newly revised Regulations on the Control of Smoking in Public Places officially took effect in Hangzhou. The regulations expand the scope of application, strictly control the scope of smoking places, adopt a multisectoral supervision model, increase smoking control measures, and improve legal responsibilities [27]. In addition, e-cigarette control has been added to the new regulations [27], which fills the legislation gap of e-cigarette control in the local tobacco regulation of China. Owing to the regulations’ significance in tobacco control of China, this study aimed to understand the public’s responses to the revision and enforcement of tobacco control regulations in China by analyzing the messages about the regulations on Sina Weibo (ie, the largest microblogging platform in China). This study combines content analysis and network analysis to understand the public’s perception and attitude toward the implementation of the regulations as well as the influence of the regulations on social media. To the best of our knowledge, this is the first study to leverage social media to analyze the impact of tobacco control regulations including e-cigarettes in China. This study will facilitate timely understanding of the impact of the implementation of the tobacco control regulations and the public’s responses to the first batch of regulation of e-cigarettes in China for public health community and policy makers.

## Methods

### Data Collection

We collected microblogs about the smoking control regulations in Hangzhou on Sina Weibo by developing web crawlers with a programming language called Python (Python Software Foundation) and a Python package called selenium [28]. The data were collected from January 1, 2019, to January 8, 2019. According to our observation, the data reached the peak on the second day and then decreased rapidly. During the week after January 8, 2019, we collected only 35 microblogs, which is only 0.44% (35/7924) of the data collected from January 1, 2019, to January 8, 2019, so the data of about 1 week were sufficient for this study. The reason for choosing Sina Weibo was that it has a large user base, with 376 million monthly active users and 165 million daily active users [29]. We first collected postings by 4 keywords, including “杭州 控烟令 (Hangzhou Smoking Control Order),” “杭州 禁烟令 (Hangzhou No Smoking Order),” “杭州 禁烟 (Hangzhou No Smoking),” and “杭州 控烟 (Hangzhou Smoking Control).” The keywords were very commonly used by Sina Weibo users in their discussions within the week after the regulation was announced. Sina Weibo has two approaches to forwarding postings. In the first approach, forwarded postings are visible to everyone; however, in the other approach, postings are only visible to some users allowed. For each relevant microblog, we also collected their reposted microblogs visible to all netizens.

We collected 7776 original microblogs by using our designed web crawlers. As to each microblog, we collected its metadata including its posted time, author name, originality, the number of times being forwarded, the number of times being liked, the number of comments, the number of times being praised, and the hyperlink of the original posting if it was not original. For some microblogs that we did not collect by web crawlers, we extracted contents and usernames based on the specific structure of their forwarding microblogs. For example, for a microblog, the user of the microblog is “Xiao Xia171,” and the content of the microblog is “Support, I hope that Anji will also upgrade the smoking ban. //@Mu Jian: Support～.” After the extraction process, we got two microblogs: “Xiao Xia171: Support, I hope that Anji will also upgrade the smoking ban” and “Mu Jian: Support～.” The second microblog “Mu Jian: Support～,” which we did not get by web crawlers, was added to our dataset. In this way, we got another 148 uncollected microblogs. In the end, we retrieved 7924 postings of 7255 users. Then, we collected the users’ profile information, including the number of followers, the number of followees, the number of posts, and the verification label, which indicates a well-known celebrity or organization.

### Content Analysis

To understand the public’s perception of the tobacco control regulations in Hangzhou, we followed the coding practice as mentioned in Harris et al [30] to code our collected 7924 postings with different stances and themes. First, the two authors read all microblogs and proposed a list of themes independently, and then they came up with the final categories of themes by discussion. Second, the two authors labeled each post with stances and themes independently and reached a consensus for all posts by discussion. Finally, we developed five themes to code the posts, including regulation-related news sharing, policy development, policy implementation, related benefits, and health and science. Each theme contained several subthemes. Each microblog was assigned only one theme and one of its subthemes. The meanings of these themes and subthemes are depicted in Table 1. In total, we proposed a list of 5 themes and 11 subthemes. The Cohen kappa statistic was used to test the reliability of coding the themes of the posts [31]. The kappa statistic was 0.88, which indicates high reliability.

To gain further understanding of the public's attitude toward the new regulations, we classified each microblog into three types of stances: Pro-Regulation (Pro-R), Anti-Regulation (Anti-R), and Discussion (Discuss). Pro-Regulation was used to describe the messages that promote the new regulations. Anti-regulation meant that the messages of this type expressed negative attitudes toward the new regulation. Any messages about the regulations that cannot be classified as either Pro-Regulation or Anti-Regulation were coded as “Discussion.” Similarly, the kappa coefficient for coding the stances was 0.90, which indicates high reliability.

**Table 1:** The description of themes and subthemes used for coding.

Themes	Description of themes	Subthemes	Description of subthemes
Regulation-related news sharing	Posting or forwarding regulation content	Regulations; discussions about the regulations	Direct posted or forwarded policy content without comments; comments on the details of the regulations
Policy development	Discussing the rationality of policy development	Scope of tobacco control; sources of production and sale	Discussion on the tobacco control scope; discussion on whether to control the sources of tobacco production and sale
Policy implementation	Effect of policy implementation and punitive measures	Effect of the policy; punishment	Discussion on the effect of policy implementation; discussion on what kind of punishment measures to use
Related benefits	Reasons for the introduction of new regulations, the impact of taxes or e-cigarettes on traditional cigarettes	E-cigarette–related benefits; tax-related benefits	E-cigarettes may affect the market of traditional cigarettes; the impact of the escalation of smoking control on taxes
Health and science	Health and scientific content related to e-cigarettes and traditional cigarettes	Addiction and quitting smoking; harms of e-cigarettes; harms of traditional cigarettes and secondhand smoke	Discussion on addiction and quitting smoking; impact of e-cigarettes on health; impact of traditional cigarettes and secondhand smoke on health

### Network Analysis

In public health, network analysis has been used to study disease transmission, information transmission, the influence of social networks on health behavior [32-36], and so on. In this study, we used network analysis and visualization methods to analyze the diffusion pattern of microblogs related to smoking control. Specifically, we constructed a transmission network in which nodes represent microblog users and edges represent reposting relationships between users. The transmission network is directed, and its direction indicates the flow of information. For example, if user B reposts a microblog of user A, the directed edge from A to B is established. In such a directed network, the outdegree centrality indicates the number of users who repost the posts of the target user. 

Highly central network members aid in the dissemination of information [37]. Therefore, we examined central network members in the transmission network to understand their role in information dissemination on smoking control. The transmission network was constructed based on the dataset of reposts we collected. The number of forwarded microblogs collected from a third-party perspective will be less than the actual number of forwarded microblogs because Sina Weibo allows users to specify some users who are allowed to read when forwarding microblogs and the unspecified users cannot see the forwarded microblogs. When we evaluated the key members in the transmission network, we considered both outdegree centrality of the transmission network and network members’ microblog forwarding number. We defined a key member as a network node whose outdegree centrality is more than 35, and the forwarding number of every microblog that this member has posted is more than 100. Most microblogs (80%) were posted or reposted by these key members.

In addition to outdegree centrality, we also measured the key original posts with the following metrics: the number of forwarded microblogs, the number of collected forwarded microblogs, effective transmission rate, effective support rate, and effective opposition rate.

The aim of the transmission network was to analyze the transmission of stance from microblog forwarding. If the collected forwarded microblogs did not contain the comment information, it could not reflect the netizens’ stance on the regulations, so it was not what we should focus on. Effective forwarded microblogs meant the forwarded microblogs we collected contained users’ support (stance “Pro-R”) or opposition (stance “Anti-R”) information. In contrast, ineffective forwarded microblogs meant the microblogs that were just forwarded without any comments or with unclear attitudes (stance “Discuss”). Effective transmission rate was defined as the number of effective forwarded microblogs divided by the number of collected forwarded microblogs. Effective supportive (opposition) rate was defined as the number of effective forwarded microblogs with stance “Pro-R” (stance “Anti-R) divided by the number of effective forwarded microblogs.

## Results

### Microblog Content

Among the 7924 microblogs, 12.85% (1018/7924) were in support of the smoking control regulations, 1.31% (104/7924) were opposed to the regulations, 84.12% (6666/7924) were neutral, and 1.72% (136/7924) were irrelevant to the regulations. Of the five themes identified, the most discussed theme was regulation-related news sharing with 83.22% (6594/7924) posts, and the least discussed theme was related benefits with only 0.98% (78/7924).

Multimedia Appendix 1 shows the summary of the collected microblogs by theme and stance. Here, we mention the most discussed subtopics for each theme. For the theme of regulation-related news sharing, there were 72.38% (5735/7924) of microblogs discussing subtopic 1, of which all posts were neutral for the Hangzhou regulations; 10.84% (859/7924) of posts were about subtopic 2, of which 59.3% (509/859) posts supported the regulations. For the theme of policy development, there were 3.41% (270/7924) of microblogs discussing subtopic 1, of which 88.9% (240/270) posts supported the regulations; 0.92% (73/7924) of posts were about subtopic 2, of which 58% (42/73) posts supported the regulations. For the theme of health and science, 2.51% (199/7924) of microblogs were about subtopic 1, of which 85.4% (170/199) were neutral to the regulations; 3.69% (292/7924) of posts were about subtopic 2, of which 71.2% (208/292) were neutral; 1.64% (130/7924) of posts were about subtopic 3, of which 50.0% (65/130) supported the regulations in Hangzhou. As to the theme policy implementation, there were 1.73% (137/7924) of microblogs discussing subtopic 1, of which 55.5% (76/137) were neutral to the regulations; 0.37% (29/7924) of posts were about subtopic 2, of which 52% (15/29) were neutral. For the theme of related benefits, 0.38% (30/7924) of microblogs discussed subtopic 1, of which 77% (23/30) were neutral; 0.61% (48/7924) of posts were about subtopic 2, of which 88% (42/48) were neutral.

In these 7924 microblogs, there were 14.75% (1169/7924) original microblogs and 85.25% (6755 /7924) forwarded microblogs. For the original microblogs, the most discussed theme was regulation-related news sharing (879/1169), and of these, 92.7% (815/879) were neutral to the regulations. Health and science (169/1169) was the second most popular topic, and of these, 89.3% (151/169) supported the regulations. As to the forwarded microblogs, the most discussed theme was also regulation-related news sharing (6594/6755), and of these, 78.89% (5202/6594) were neutral. The average number of posts for one Weibo user was 1.1, and the user with the most posts was Lawyer Zhiming Zhuang. Lawyer Zhuang’s views on the Hangzhou regulations were mixed, but the theme he participated in was mainly policy development.

### Transmission Network

**Figure 1:**
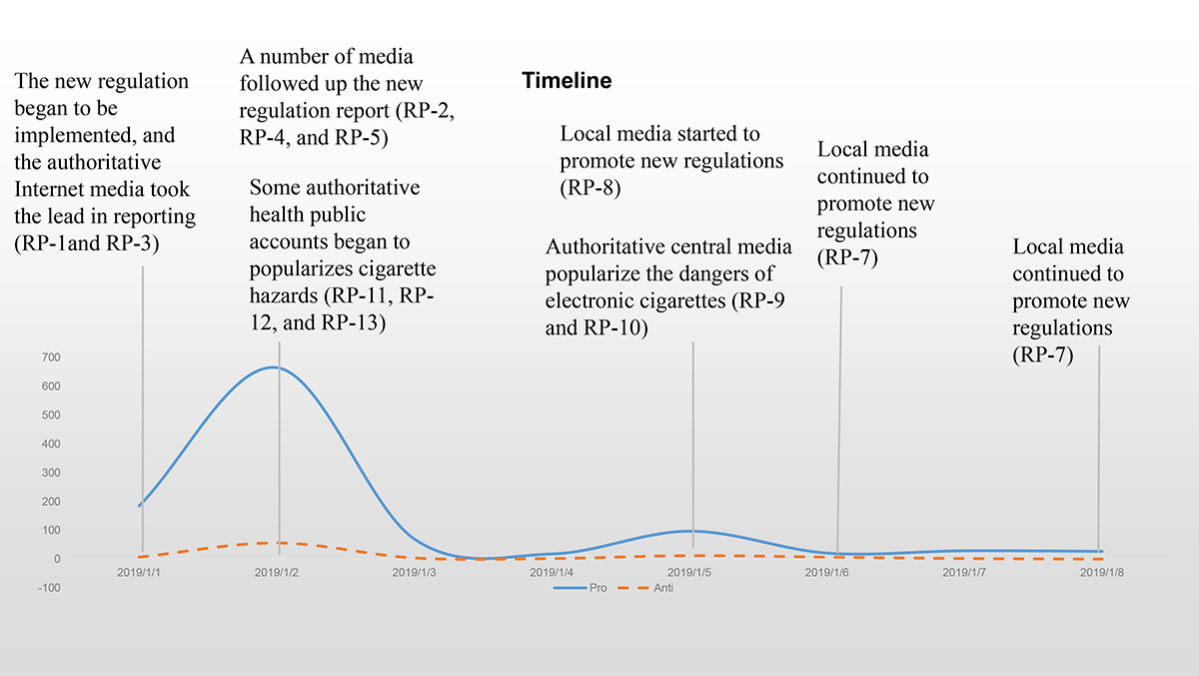
Timeline showing the distribution of antipolicy, propolicy microblogs, and repost microblogs.

The distribution of antipolicy and propolicy microblogs and repost microblogs is shown in Figure 1. Most microblogs were sent between January 1, 2019, and January 2, 2019. The propolicy microblogs and antipolicy microblogs peaked on January 2, but the former were far greater than the latter. Throughout the week, there were fewer antipolicy microblogs and repost microblogs.

According to the aforementioned network analysis method, 12 key accounts involving 13 key original microblogs were extracted from the data, named RP-1 to RP-13. Among them, account “@CCTV News” had two related original microblogs (RP-2 and RP-9). Table 2 shows the 13 key original microblogs, and detailed content is presented in Table 3. The new regulation was implemented, starting January 1, 2019, and the authoritative internet media took the lead in reporting (RP-1 and RP-3). On the next day, several media agencies followed up the new regulation report (RP-2, RP-4, and RP-5), and some authoritative health public accounts began to popularize cigarette hazards (RP-11, RP-12, and RP-13), which led to a lot of discussion. After a few days, local media started to promote new regulations (RP-8), and authoritative central media popularized the dangers of e-cigarettes (RP-9 and RP-10) on January 5, 2019, which brought the second peak of discussion. Then, local media continued to promote new regulations (RP-7 and RP-8) until January 8, 2019. During the week after January 8, 2019, we collected only 35 microblogs, which is only 0.44% (35/7924) of the data collected from January 1, 2019, to January 8, 2019, so we did not analyze the data after January 8, 2019.

**Table 2:** Summary of the key original microblogs.

Categories, type of account, and account^a^			Content	Collected forwarded rate, n/N (%)^b^	Effective transmission rate, n (%)^c^	Effective support rate, n (%)^d^	Effective opposition rate, n (%)^d^
**Regulation-related news sharing**							
	**News media**						
		The Paper	RP-1	1651/4584 (36.02)	190 (11.51)	178 (93.7)	12 (6.3)
		CCTV News	RP-2	414/469 (88.3)	151 (36.5)	148 (98.0)	3 (2.0)
		NewsHead	RP-3	237/262 (90.5)	94 (39.7)	87 (93)	7 (7)
		People's network	RP-4	157/165 (95.2)	32 (20.4)	29 (91)	3 (9)
		China News Network	RP-5	87/111 (78.4)	21 (24)	18 (86)	3 (14)
		Total	N/A^e^	2546/5591 (45.54)	488 (19.17)	460 (94.3)	28 (5.7)
	**Local news media**						
		Hangzhou’ s Big Popular Life	RP-6	155/1221 (12.69)	21 (13.5)	21 (100)	0 (0)
		Hot Events in Hangzhou	RP-7	97/1270 (7.64)	0 (0)	0 (0)	0 (0)
		Hangzhou Information Headlines	RP-8	45/952 (4.72)	1 (2)	1 (100)	0 (0)
		Total	N/A	297/3443 (8.63)	22 (7.3)	22 (100)	0 (0)
**Health promotion**							
	**News media**						
		CCTV NEWS	RP-9	1120/1315 (85)	104 (9.29)	86 (82.7)	18 (17.3)
	**Local news media**						
		Hangzhou Top Information List	RP-10	122/1034 (12)	1 (0.8)	0 (0)	1 (100)
	**Self-media Weibo accounts**						
		Clove doctor	RP-11	470/593 (79.3)	62 (13.2)	55 (89)	7 (11)
		Health preservation - Lao Yang	RP-12	37/133 (27.8)	1 (3)	1 (100)	0 (0)
		Rice cake mother	RP-13	73/112 (65.2)	3 (4)	3 (100)	0 (0)
		Total	N/A	580/838 (69.2)	66 (11.4)	59 (89)	7 (11)

^a^See Multimedia Appendix 2 for the Weibo usernames in Chinese and English.

^b^The N value for each microblog reflects the number of retweets.

^c^The denominator of the n values is the corresponding n value of the Collected forwarded rate column.

^d^The denominator of the n values is the corresponding n values of the Effective transmission rate column.

^e^N/A: not applicable.

**Table 3:** The detailed content.

Number of post	Content^a^
RP-1	Hangzhou is Upgrading Smoking Control Ordinance: Electronic cigarettes are included in the scope of smoking ban; the maximum penalty for illegal smoking is 20,000 RMB. The newly revised Hangzhou Regulations on Smoking Control in Public Places was formally implemented on January 1. Hangzhou is one of the earliest cities in China to carry out tobacco control legislation, and tobacco control work has been steadily and firmly promoted. the latest “smoking control order” has expanded the scope of regulation, strictly controlled the scope of smoking places, and put forward the requirements of smoking ban in outdoor areas of some public places. the implementation of a multi-sectoral supervision model has strengthened law enforcement. electronic cigarettes are also included in the smoking ban. If smokers are found to be smoking in non-smoking areas, they may be asked to stop smoking or leave the place immediately. If not dissuaded, citizens can call “12345” to report complaints. after registering and accepting complaints, the cases will be handed over to the corresponding tobacco control regulatory authorities for disposal in accordance with the division of duties of tobacco control supervision.
RP-2	Hangzhou Smoking Control Ordinance: Electronic cigarettes are included in the smoking ban with a maximum penalty of 20,000 RMB. The newly revised Hangzhou Regulations on Smoking Control in Public Places were formally implemented on January 1. it is clearly stipulated that smoking is prohibited in indoor public places, indoor workplaces and public transport. therefore, smoking is prohibited even in one's own office. smoking places are prohibited not only from lighting tobacco products and smoking traditional cigarettes, but also from smoking electronic cigarettes.
RP-3	Hangzhou Upgrading Smoking Control Ordinance: Electronic cigarettes are included in the scope of smoking ban, the maximum penalty for illegal smoking is 20,000 RMB. The newly revised Hangzhou Regulations on Smoking Control in Public Places was formally implemented on January 1. the latest “smoking control order” has expanded the scope of application, strictly controlled the scope of smoking places, and put forward the requirements of smoking ban in outdoor areas of some public places. the implementation of a multi-sectoral supervision model has strengthened law enforcement. electronic cigarettes are also included in the smoking ban.
RP-4	Hangzhou Upgrading Smoking Control Ordinance: Electronic cigarettes were included in the smoking ban with a maximum penalty of 20,000 RMB. The newly revised Hangzhou Regulations on Smoking Control in Public Places were formally implemented on January 1. the latest “smoking control order” has set a buffer period for smoking places. electronic cigarettes are included in the scope of smoking ban. if operators and managers fail to fulfill their duties of smoking control, they can be fined up to 20,000 yuan.
RP-5	Hangzhou Upgraded Smoking Control Ordinance: Electronic cigarettes were included in the smoking ban, with a maximum penalty of 20,000 RMB for illegal smoking. The newly revised Hangzhou Regulations on Smoking Control in Public Places was formally implemented on January 1. the latest “smoking control order” has expanded the scope of application, strictly controlled the scope of smoking places, and put forward the requirements of smoking ban in outdoor areas of some public places. the implementation of a multi-sectoral supervision model has strengthened law enforcement. electronic cigarettes are also included in the smoking ban.
RP-6	Hangzhou Upgrading Smoking Control Ordinance: Electronic cigarettes were included in the smoking ban with a maximum penalty of 20,000 RMB. The newly revised Hangzhou Regulations on Smoking Control in Public Places were formally implemented on January 1. The latest “smoking control order” has set a buffer period for smoking places. electronic cigarettes are included in the scope of smoking ban. if operators and managers fail to fulfill their duties of smoking control, they can be fined up to 20,000 yuan.
RP-7	# Hangzhou Hot Events # The newly revised Regulations on Smoking Control in Public Places of Hangzhou have been formally implemented. the latest “smoking control order” has expanded the scope of application, strictly controlled the scope of smoking places, and put forward the requirements of smoking ban in outdoor areas of some public places. the implementation of a multi-sectoral supervision model has strengthened law enforcement. electronic cigarettes are also included in the smoking ban.
RP-8	Hangzhou Upgrading Smoking Control Ordinance: Electronic cigarettes are included in the scope of prohibition, with a maximum penalty of 20,000 RMB for illegal smoking. The newly revised Hangzhou Regulations on Smoking Control in Public Places have been formally implemented. the latest “smoking control order” has expanded the scope of application, strictly controlled the scope of smoking places, and put forward the requirements of smoking ban in outdoor areas of some public places. the implementation of a multi-sectoral supervision model has strengthened law enforcement. electronic cigarettes are also included in the smoking ban.
RP-9	This kind of smoke is harmful! The newly revised Hangzhou Regulations on Smoking Control in Public Places came into effect on January 1. no smoking places not only prohibit lighting tobacco products and smoking traditional cigarettes, but also prohibit smoking electronic cigarettes. why are electronic cigarettes banned?
RP-10	# Hangzhou Headline. This kind of smoke, harmful! The newly revised Hangzhou Regulations on Smoking Control in Public Places came into effect on January 1. no smoking places not only prohibit lighting tobacco products and smoking traditional cigarettes, but also prohibit smoking electronic cigarettes. why are electronic cigarettes banned?
RP-11	Smoking is harmful to health. what about electronic cigarettes? Let’s do a science popularization. # Hangzhou electronic cigarettes are included in the smoking ban#
RP-12	# Hangzhou electronic cigarettes are included in the smoking ban# A video tells you how harmful smoking is. after watching it, everyone decides to quit smoking. [Health preservation—Lao Yang’s Weibo Video Link] @ Health preservation—LaoYang
RP-13	# Hangzhou electronic cigarettes are included in the smoking ban# Comprehensive tobacco control from family to society is the right way for parents to protect their children from second-hand smoke. smoke-free and healthy environment is the best gift we give our children. [Rice cake mother's Second Shot Video Link] @ Rice cake mother

^a^See Multimedia Appendix 3 for the detailed content in Chinese and English.

The microblogs in Table 2 can be divided into two themes: regulation-related news sharing and health. There were 8 microblogs about “regulation-related news sharing,” and the news media and local news media accounts were the main communication accounts. For the microblogs about “regulation-related news sharing,” the effective transmission rate of news media was high, and the effective support rate was high. The effective transmission rate of local news media was very low, but the effective support rate was high. This may be because the audiences of the local news media were mostly local users, and they were more inclined to use the comment function as a means of chatting with friends. The content of the comment was mostly the content of chatting between friends who did not want the content to be seen by others, so the effective transmission rate was low. There were 5 microblogs about health, and the corresponding accounts were news media, local news media, and self-media Weibo accounts. These microblogs were mainly about the dangers of e-cigarettes (RP-9, RP-10, and RP-11) and the harms of traditional cigarettes (RP-12) and secondhand smoke to young people (RP-13). The total effective transmission rate of these microblogs was medium, but the total effective opposition rate was high. Especially with regard to the health hazards of e-cigarettes (RP-9, RP-10, and RP-11), their opposition position was significantly higher than the other two microblogs (RP-12 and RP-13) in the same theme, resulting in high total effective opposition rate of the theme “health,” which reflects the great differences among netizens in the impact of e-cigarettes on health.

The transmission network consisted of 6436 Weibo users who reposted at least one post from other users, and 819 isolated users who did not repost posts. There were 6330 asymmetric ties and 22 mutual ties in the network. A user usually has 1 to 3 other users who repost his or her posts, except for user “Lawyer Zhiming Zhuang” with 27 users reposting his posts.

**Figure 2:**
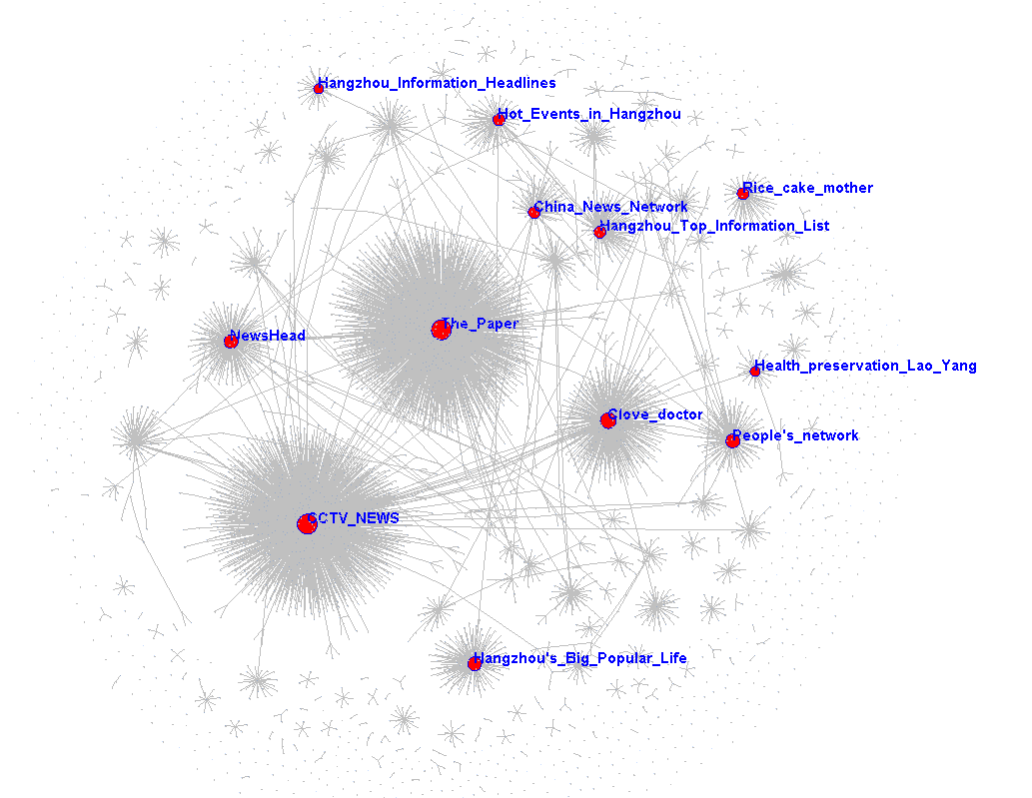
Transmission network with nodes sized by outdegree and the 12 key Weibo users shown in red.

Figure 2 shows the transmission network where node size represents outdegree: the larger a node, the higher the frequency of its posts being reposted. In the transmission network, there were 12 key Weibo users with 13 key original microblogs.

Microblogs RP-1 to RP-5 were related to a newspaper article detailing the differences between the latest antismoking policy and previous policies. It elaborated the buffer period to construct suitable smoking rooms for customers, multisector supervision mode, prohibition of e-cigarettes, the scope of smoking places (including indoor public places, indoor workplaces, public transportation, and some outdoor areas), and so on. Owing to the relatively earlier time of publication and rich content, the number of reposts reached 5591. The main categories of the reposted microblogs included discussion of the regulations, scope of tobacco control, effect of the policy, and sources of production and sale.

Microblogs RP-6 and RP-8 outlined the significant differences between the latest antismoking policies and previous policies. Their accounts were the local information accounts. The total number of forwarding posts was 3473, and the number of collected forwarding posts was 300, but the effective rate was only 7%.

Microblogs RP-9 to RP-10 focused on the e-cigarettes mentioned in the regulations by using long images. Referring to a large amount of survey data, the microblogs illustrated the definition of e-cigarettes, the differences between e-cigarettes and combustible cigarettes, and whether e-cigarette use was really “harmless.” They also discussed that e-cigarettes should not be ignored and that e-cigarettes should be used to assist in quitting smoking according to doctors’ advice. The main categories of repost microblogs included harms of e-cigarettes, discussion of the regulations, taxes, harms of traditional cigarettes and secondhand smoke, addiction and quitting smoking, places and scopes of tobacco control, and interest issues. Microblog RP-11 contained a long image that referred to a journal article related to e-cigarette policy published by the American Heart Association. This microblog outlined the regulatory status of e-cigarettes around the world and analyzed in detail the composition of e-cigarettes and their health harms, which was reposted 593 times. The main categories of these repost microblogs were as follows: harms of e-cigarettes, discussion of the regulations, addiction and quitting smoking, and tobacco ban and quitting smoking.

Microblog RP-12 contained a video that simulated the health hazards of direct and secondhand smoke through an experiment. The microblog was reposted 133 times. The main category was harms of traditional cigarettes. Microblog RP-13 contained a Web hyperlink to a video about interviewing innocent children with regard to their views on secondhand smoking. It was reposted 112 times.

## Discussion

### Principal Findings

When the tobacco control regulations in Hangzhou were promoted on social media, the content of the regulations was highlighted by different types of social media accounts from several angles: the expansion of the regulation scope, the expansion of the scope of smoking control, the definition of multisectoral supervision, and the inclusion of e-cigarettes. For the temporal distribution of the collected data, the revised smoking control legislation took effect in Hangzhou on January 1, and the discussion peaked on January 2, when people began to rationally analyze the reasons and effects for smoking control.

Within a few days after the publication of the regulations, several discussions about the regulations took place around several major accounts. Most users were just involved in the forwarding and discussion of the regulations (6119/7255, 84.34%), without explicit support or opposition. Among the 1043 users who explicitly expressed their support or opposition to the policy, a large proportion of users showed supportive attitudes (956/1043, 91.66%). Among the 965 users who showed supportive attitude, 273 users expressed their views on policy development. Some thought that the scope of the regulations should be extended to other cities, and some people thought that tobacco sales should be included in the regulations. A total of 480 users participated in the discussion of the regulations. They mainly focused on and supported three aspects of the regulations. First, the regulations strictly control the scope of tobacco control and clearly stipulate that smoking is prohibited in indoor public places, indoor workplaces, and public transportation. They are supported by most Weibo users. For example, one user posted, “No Smoking in indoor workplaces.” Second, the regulations also impose a smoking ban on outdoor areas in some public places. They also save people from the pain of passive smoking in some places outside. Another user posted, “The smoking ban in Hangzhou is worthy of praise! The regulations also impose smoking bans on outdoor areas in some public places.” Third, the regulations have incorporated e-cigarettes into the smoking ban and have received support from many users who hate e-cigarettes. For instance, a user posted, “In public, smoking e-cigarettes is also inappropriate. Support the ban on all cigarettes in public.”

In terms of topics of concern to netizens, as shown in the microblog content analysis results and Multimedia Appendix 1, Weibo users were generally concerned about (including support, opposition and discuss) policy implementation (n=165) and the impact of smoking and secondhand smoke on health (n=130). In particular, because of the introduction of e-cigarettes into the scope of supervision for the first time, netizens were also concerned about the hazards of e-cigarettes (n=286). Some Weibo users, especially users in Beijing and Guangzhou, expressed their hope that the policy could be extended to their cities (n=270) when forwarding microblogs.

The public needs to raise awareness about the motivations behind the regulations. Some netizens were worried that the tobacco control regulations would accelerate the increase in the tobacco tax rate, which would lead to an increase in the price of tobacco products, even though China's tobacco tax is far below the average in the world [38]. Therefore, to solve these concerns, more effort is needed to promote and explain the tobacco control regulations through social media.

The public health community and the government need to put more effort into popularizing the knowledge about e-cigarettes to enhance the public’s acceptance of e-cigarette control. Although many netizens believe that smoking leads to a health hazard, some people oppose the control of e-cigarettes and believe that e-cigarettes are not harmful at all or less harmful than cigarettes (n=13). Some netizens (n=73) believe that the control of e-cigarettes was added to the regulations because of the interest divergence between e-cigarettes and traditional cigarettes. They also believe that the government intends to impose high taxes on e-cigarettes, so they first introduced e-cigarette control in the regulatory policies.

The public needs to increase their trust in the enforcement of the regulations. Some users believe that the regulation is well formulated, but the most important thing is the implementation effect of the regulation. Some netizens (n=76) criticized that the previous smoking control regulations were ineffective, and the regulatory measures did not take effort. This reflects the fact that although Chinese netizens generally support the smoking control regulations in public places, they distrust the effectiveness of the corresponding policy implementation. Therefore, the government should pay attention not only to the rationality of policy formulation but also to the actual effect of policy implementation. In addition, policy makers can use social media to gain feedback on policy implementation to improve the quality of tobacco control.

### Limitations

Some limitations should be considered when interpreting the results. First, more than 80% of Sina Weibo users are under the age of 30 years. The number of male users (56.3%) is slightly higher than that of female users (43.7%). Users from third- and fourth-tier cities account for more than 50% of Weibo’s monthly active users [29]. Although our data were collected from the most popular social media platforms, further research is needed on other social media platforms in China. Second, our data only cover the first week after the regulations were issued in Hangzhou. On the basis of the temporal distribution of the collected data, the discussions about the regulations decreased gradually after the first peak, so the data were still reasonable for this study although the time interval was not very long. Third, the tobacco control regulations mentioned in this paper were issued by the Hangzhou government, so the regulations’ influence is still limited because they are not a national policy.

### Conclusions

Given the very high prevalence of smoking in China, reducing smoking and tobacco use is difficult because it requires both policy changes and changes in social norms. The recent tobacco control policy in Hangzhou led to a heated discussion on social media, and we conducted this study to understand how the public responded to the impact of the new policy on consumers, tobacco industry, and the public interest. We found that the number of posts supporting the policy were significantly higher than those that were against the policy. The topics of these posts were diverse, including regulation scope, health effects of cigarettes and e-cigarettes, taxes, policy enforcement, and so on. We also found that most of the key social media accounts in spreading the policy were professional new media accounts rather than individual accounts. Our findings demonstrate the possibility of using social media to evaluate the influence of public health policy and understand public perceptions. As the use of social media grows, more research is needed to better understand how to develop and implement sound tobacco control policies and relevant campaigns to engage the public to improve their health.

## Appendix

Multimedia Appendix 1 The original text of Microblogs.

Multimedia Appendix 2 Weibo usernames in Chinese and English.

Multimedia Appendix 3 Chinese-English contrast table of microblog contents.

## Abbreviations

e-cigarette: electronic cigarette
NSFC: National Natural Science Foundation of China
WHO: World Health Organization
