# Supplementary material for: Public Reactions to the Cigarette Control Regulation on a Chinese Microblogging Platform: Empirical Analysis
Source: J Med Internet Res. 2020 Apr 27;22(4):e14660. doi: 10.2196/14660 (PMC7215491; doi:10.2196/14660)
Supplement: Multimedia Appendix 1 [file jmir_v22i4e14660_app1.docx]

**Appendix 1**

Summary of the responses to Hangzhou’s new regulations on smoking control on social media.

| **Topic** | **Subtopic** | **Stance** | **All n=7924** | **Example** |
| --- | --- | --- | --- | --- |
| **Regulation-related news sharing** | Regulations | Pro-R | 0 (0.00%) |  |
|  |  | Anti-R | 0 (0.00%) |  |
|  |  | Discuss | 5735 (72.38%) | ... but [they] also prohibit smoking e-cigarettes. Why are e-cigarettes banned? ... ​​​ |
|  | Discussions about the regulations | Pro-R | 509 (6.42%) | No Smoking in indoor workplaces |
|  |  | Anti-R | 68 (0.86%) | E-cigarettes use water vapor and have nothing to do with public health. It is fool to include e-cigarettes in the regulation.. |
|  |  | Discuss | 282 (3.56%) | With the upgrade of Hangzhou's smoking control regulations, … Such an approach has aroused heated discussions among passengers. |
| **Policy development** | Scope of tobacco control | Pro-R | 240 (3.03%) | Recommended national promotion |
|  |  | Anti-R | 5 (0.06%) | If you want to ban smoking, can you set up smoking rooms everywhere just like what Japan government does?? |
|  |  | Discuss | 25 (0.32%) | It is necessary to have a closed smoking room nationwide and only smoke in the smoking room. ... |
|  | Sources of production and sale | Pro-R | 42 (0.53%) | The state should set a timetable for the withdrawal of the tobacco industry (agriculture) as soon as possible... |
|  |  | Anti-R | 5 (0.06%) | Don't sell cigarettes at all. |
|  |  | Discuss | 26 (0.33%) | Why not start from the source? ​ |
| **Health & science** | Addiction and quitting smoking | Pro-R | 17 (0.21%) | ... I don't smoke, I may not understand what these people think. The cigarette addiction is so severe. |
|  |  | Anti-R | 2 (0.03%) | It is definitely not a good thing for smokers to quit smoking. Once they stop smoking, the probability of suffering from ulcerative colitis is more than 70% higher than that of people who do not smoke. In addition ... |
|  |  | Discuss | 170 (2.15%) | … Why is smoking addictive? What are the ways to quit smoking? |
|  | Harms of E-cigarette | Pro-R | 65 (0.82%) | …if you really want to be healthier, just honestly quit smoking. Don't fool yourself. |
|  |  | Anti-R | 13 (0.16%) | The dangers of e-cigarettes have been seen from relevant literature and experimental programs supported by the United States … For my dad, I learned a lot of knowledge about e-cigarettes and persuade him to use e-cigarettes… |
|  |  | Discuss | 208 (2.62%) | ... Are these e-cigarettes known as “helping to quit smoking”, “healthy and harmless,” and “tar-free” really “healthy and harmless?” … |
|  | Harms of traditional cigarettes and secondhand smoke | Pro-R | 65 (0.82%) | Support, …, I hope that we can implement it as soon as possible! |
|  |  | Anti-R | 1 (0.01%) | Avoid talking about the tar that is the biggest hazard in cigarettes, the whole article is saying that electronic cigarettes may have high nicotine and that additives may be at risk, and then come to the conclusion that electronic cigarette are more harmful?... |
|  |  | Discuss | 64 (0.81%) | This is theoretically the case, but quitting smoking is not necessarily healthier... |
| **Policy implementation** | Effect of the policy | Pro-R | 60 (0.76%) | I hope that the supervision can keep up with it. …protect the implementation of policies. |
|  |  | Anti-R | 0 (0.00%) |  |
|  |  | Discuss | 76 (0.96%) | …Some people say that it's finally up to you, but others disapprove of it. … |
|  | Punishment | Pro-R | 13 (0.16%) | Strict punishment is better than vigorous prohibition of smoking. Public morality should be observed in public places. You can do anything, but listen to dissuasion. Don’t do something that people around you oppose… |
|  |  | Anti-R | 1 (0.01%) | Comparing to the legal punishment for stealing, this is a bit too heavy. |
|  |  | Discuss | 15 (0.19%) | We should learn from other areas, such as the Macao Special Administrative Region, where the maximum fine for smoking in public places is Macau$1500. ​ |
| **Related benefits** | E-cigarette–related benefits | Pro-R | 4 (0.05%) | …this is the fundamental interests of the government for tobacco. Hangzhou goes ahead as always. … |
|  |  | Anti-R | 3 (0.04%) | After the tobacco company developed its own e-cigarettes, it became a healthier product with low tar content. |
|  |  | Discuss | 23 (0.29%) | I also think that e-cigarettes have harmed interests of the tobacco company. |
|  | Tax-related benefits | Pro-R | 1 (0.01%) | It has nothing to do with taxes. Smoking in public places is uncivilized. |
|  |  | Anti-R | 5 (0.06%) | Of course, they can't ban smoking, because the big tobaccos can pay trillions of taxes every year. |
|  |  | Discuss | 42 (0.53%) | I think this is mainly due to tax failure. |

The original text of Microblogs.

| **Topic** | **Subtopic** | **Stance** | **All n=7924** | **Example** |
| --- | --- | --- | --- | --- |
| **Regulation-related news sharing** | Regulation | Pro-R | 0(0.00%) |  |
|  |  | Anti-R | 0(0.00%) |  |
|  |  | Discuss | 5735(72.38%) | 1日，最新修订的《杭州市公共场所控制吸烟条例》正式实施。禁烟场所不仅禁止点燃烟草制品和吸传统卷烟，也禁止吸电子烟。电子烟为何会被明令禁止？戳图了解↓↓转给身边吸烟的TA！ ​​​ |
|  | discuss of Regulation | Pro-R | 509(6.42%) | 室内工作场所禁止吸烟 |
|  |  | Anti-R | 68(0.86%) | 电子烟吐的是水蒸汽碍着谁了没事就干这些一刀切的沙雕规定 |
|  |  | Discuss | 282(3.56%) | 随着杭州控烟令的升级，从元旦开始，杭州萧山机场国内和国际航站楼内的吸烟室都关闭了，结束了18年的吸烟室历史。这样的做法引起旅客的热议。 L浙样红TV的微博视频 ​ |
| **Policy development** | scope of control | Pro-R | 240(3.03%) | 建议全国推广 |
|  |  | Anti-R | 5(0.06%) | 要这么禁烟可以，倒是像日本一样到处都有的吸烟点吸烟室配置跟上啊？？一刀切禁了就不管了？ |
|  |  | Discuss | 25(0.32%) | 就应该全国范围设封闭式的吸烟室，只许在吸烟室内吸烟，其他任何地方，室内和大街上都严禁吸烟。 |
|  | source of cigarette product and sale | Pro-R | 42(0.53%) | 国家应该尽快制定烟草工业（农业）退出时间表，让人民看到国家禁言的诚意和决心，同时给予相关企业和人员转产转业的过渡期，在烟草产品收入中预留部分将来用作安置的费用。 |
|  |  | Anti-R | 5(0.06%) | 有本事不要卖烟 |
|  |  | Discuss | 26(0.33%) | 为什么不从源头开始禁呢 ​ |
| **Healthy&science** | addiction and quit smoking | Pro-R | 17(0.21%) | 昨天在星巴克，营业员连续劝阻了俩抽电子烟的年轻人。我不抽烟，可能不能体会这些人是怎么想的，烟瘾这么大，买咖啡的功夫都忍不住吗？ |
|  |  | Anti-R | 2(0.03%) | 吸烟的人戒烟绝对不是好事，一但停止吸烟，患上溃疡性结肠炎的概率比平时不吸烟的人高70%以上。另外，吸烟的人患上结肠炎概率比不吸烟的人低2-5倍从八十年代起全球就讨论研究过这个现象，医院里因结肠炎住院的人很少是吸烟者，要相信科学控就控吧，别把自己搞成恐怖分子一样，赶尽杀绝，首都机场连吸烟室也不设 收起全文d |
|  |  | Discuss | 170(2.15%) | 所谓吸烟成瘾，在我看来就是个生活习惯问题。几十年了，我从不敢妄称自己是烟民。因为，拿起笔或端起酒杯来，我习惯一根接一根地抽烟；周六周日在家鼓捣点小活，我可以把吸烟的事忘掉。对我来说，烟可以说抽就抽，说扔就扔，从来就不存在障碍。连我自己都纳闷，这算几等烟民呐？！ O为什么抽烟会上瘾呢？戒烟的方法有哪些？ 收起全文d |
|  | harm of E-cigarette | Pro-R | 65(0.82%) | 电子烟也是有害身体健康的，什么无害的XXX电子烟，什么天然烟油健康清肺，都是商家忽悠你的要是真的想要健康，还是老老实实戒烟吧，别自欺欺人了 |
|  |  | Anti-R | 13(0.16%) | 电子烟的危害美国已经有相关文献和实验节目可以看到了，危害与每天喝咖啡差不多，电子烟的烟雾是蒸汽，卷烟的烟雾才是危害性极大地二手烟，既然不禁售烟草，那么两者相比，电子烟更应该被推广和管控质量，尼古丁多大剂量才能让人中毒？为了老爸我做了很多功课给他换了电子烟，说害人，哪个害人没B数？ |
|  |  | Discuss | 208(2.62%) | 1日， 电子烟也被纳入禁烟范围，违法吸烟最高罚2万。随着我国多个城市实施禁烟令以来，一种加热不燃烧的低温卷烟，俗称iQOS电子烟，因其外观时尚，烟雾小等特点，逐渐年轻人的青睐。这些号称“帮助戒烟”“健康无害”“不含焦油”的电子烟真的“健康无害”吗？一份检测报告显示，这种加热不燃烧卷烟的电子烟，不仅同样含有尼古丁（即烟碱），而且含有亚硝胺等致癌物！ L央视财经的酷燃视频 L央视财经的酷燃视频 收起全文d |
|  | harm of traditional cigarettes and secondhand smoke | Pro-R | 65(0.82%) | 支持，这些年被二手烟熏的脑袋疼，闻二手烟喉咙疼老是搞的我喉咙发炎，胃泛酸拉肚子，长此以往我真担心得癌，真的该好好抓抓了，凭什么我就要被迫接受这些荼毒！希望我们这也赶快实施起来！ |
|  |  | Anti-R | 1(0.01%) | 对香烟中最大的危害焦油避而不谈，全篇充斥着电子烟可能存在尼古丁含量高、添加剂可能有风险，然后得出电子烟危害更大的结论？？？ 这样的博主，pink了 |
|  |  | Discuss | 64(0.81%) | 理论上是这样的，但是不吸烟的也未必就更健康。目前的研究，只能证明吸烟和这些疾病的有高相关。并不能证明是因果关系，也就是说吸烟未必一定导致这些疾病 |
| **Policy Implementation** | effect of the policy | Pro-R | 60(0.76%) | 希望监管能跟得上另外堵不如疏，在严管的同时多设立专门的吸烟场所才能更好的保障政策的施行 |
|  |  | Anti-R | 0(0.00%) |  |
|  |  | Discuss | 76(0.96%) | 2019年1月1日，号称史上最严的杭州新版禁烟令实施。新版控烟条例中有明确规定：室内工作场所、室内公共场所和公共交通工具内禁止吸烟。杭州有两家单位因禁烟不力而被罚款。有人说，终于等到你，但也有人对此不以为然。O网页链接 ​ |
|  | punishment | Pro-R | 13(0.16%) | 大力禁烟不如严格惩处，公共场合要遵守公德才行～你可以干任何事情，但是要听从劝阻，周边有人反对的事情任谁也不能干，否则后果自负！​ |
|  |  | Anti-R | 1(0.01%) | 从企业偷排处罚比例来看，这个有点过重了。 |
|  |  | Discuss | 15(0.19%) | 应该学习其他地区，比如澳门特别行政区，公共场所吸烟最高罚款1500澳门币。 ​ |
| **Benefits related** | e-cigarette related benefits | Pro-R | 4(0.05%) | “吸烟有害健康，但有利于国家”，19万亿总财政收入，中国烟草总公司上交财政1.1万亿，这是政府对于烟草的根本利益所在，杭州一如既往的走在前面。戒烟的第三个月加油 |
|  |  | Anti-R | 3(0.04%) | 等烟草公司研制出自己电子烟后就成了保健品了焦油含量底呀 |
|  |  | Discuss | 23(0.29%) | 我也觉得是电子烟动了烟草公司的奶酪。 |
|  | tax related benefits | Pro-R | 1(0.01%) | 这跟税收没关系，公共场所吸烟是不文明行为 |
|  |  | Anti-R | 5(0.06%) | 当然不能禁烟了，岁供上万亿税收呢。 |
|  |  | Discuss | 42(0.53%) | 偶觉得这主要是税收上不去。 |
