# Supplementary material for: Public Reactions to the Cigarette Control Regulation on a Chinese Microblogging Platform: Empirical Analysis
Source: J Med Internet Res. 2020 Apr 27;22(4):e14660. doi: 10.2196/14660 (PMC7215491; doi:10.2196/14660)
Supplement: Multimedia Appendix 2 [file jmir_v22i4e14660_app2.docx]

**Appendix 2**

Weibo Usernames in Chinese and English

English name Chinese name

The Paper 澎湃新闻

CCTV NEWS 央视新闻

NewsHead 头条新闻

People's network 人民网

China News Network 中国新闻网

Hot Events in Hangzhou 杭州热门大事件

Hangzhou Top Information List 杭州头条资讯榜

Hangzhou Information Headlines 杭州资讯头条

Hangzhou's Big Popular Life 杭州生活大热门

Clove doctor 丁香医生

Health preservation - Lao Yang 健康养生-老杨

Rice cake mother 年糕妈妈
