# Supplementary material for: Public Reactions to the Cigarette Control Regulation on a Chinese Microblogging Platform: Empirical Analysis
Source: J Med Internet Res. 2020 Apr 27;22(4):e14660. doi: 10.2196/14660 (PMC7215491; doi:10.2196/14660)
Supplement: Multimedia Appendix 3 [file jmir_v22i4e14660_app3.docx]

**Appendix 3**

Chinese-English Contrast Table of Microblog Contents

RP-1

English version

Hangzhou is Upgrading Smoking Control Ordinance: Electronic cigarettes are included in the scope of smoking ban; the maximum penalty for illegal smoking is 20,000 RMB.

the newly revised Hangzhou Regulations on Smoking Control in Public Places was formally implemented on January 1. Hangzhou is one of the earliest cities in China to carry out tobacco control legislation, and tobacco control work has been steadily and firmly promoted. The latest "smoking control order" has expanded the scope of regulation, strictly controlled the scope of smoking places, and put forward the requirements of smoking ban in outdoor areas of some public places. The implementation of a multi-sectoral supervision model has strengthened law enforcement. Electronic cigarettes are also included in the smoking ban. If smokers are found to be smoking in non-smoking areas, they may be asked to stop smoking or leave the place immediately. If not dissuaded, citizens can call "12345" to report complaints. After registering and accepting complaints, the cases will be handed over to the corresponding tobacco control regulatory authorities for disposal in accordance with the division of duties of tobacco control supervision.

Chinese version

【#杭州升级控烟令#：电子烟被纳入禁烟范围，违法吸烟最高罚2万】1日，最新修订的《杭州市公共场所控制吸烟条例》正式实施。杭州是国内较早开展控烟立法的城市之一，控烟工作一直持续扎实推进。此次最新“控烟令”扩大了适用范围、严格了控制吸烟场所范围、对一些公共场所的室外区域也提出了禁烟要求。实行多部门监管模式，增强了执法力量。电子烟也被纳入禁烟范围。如果发现有人吸烟，确定该场所属于禁烟场所后，可要求吸烟者立刻停止吸烟或者离开该场所。如果不听劝阻，市民可以拨打“12345”投诉举报，登记受理投诉后将按照控烟监管职责分工，移交相应的控烟监管部门处置。浙江24小时客户端 [URL][图片] @澎湃新闻

RP-2

English version

Hangzhou Smoking Control Ordinance: Electronic cigarettes are included in the smoking ban with a maximum penalty of 20,000 RMB

The newly revised Hangzhou Regulations on Smoking Control in Public Places were formally implemented on January 1. It is clearly stipulated that smoking is prohibited in indoor public places, indoor workplaces and public transport. Therefore, smoking is prohibited even in one's own office. Smoking places are prohibited not only from lighting tobacco products and smoking traditional cigarettes, but also from smoking electronic cigarettes.

Chinese version

【杭州控烟令来了：电子烟被纳入禁烟范围 最高罚2万】1日，最新修订的《杭州市公共场所控制吸烟条例》正式实施。明确规定室内公共场所、室内工作场所、公共交通工具内禁止吸烟，因此，即使在自己的办公室吸烟，也是被禁止的。禁止吸烟场所不仅禁止点燃烟草制品和吸传统卷烟，也禁止吸电子烟。O网页链接 收起全文d

RP-3

English version

Hangzhou Upgrading Smoking Control Ordinance: Electronic cigarettes are included in the scope of smoking ban, the maximum penalty for illegal smoking is 20,000 RMB.

The newly revised Hangzhou Regulations on Smoking Control in Public Places was formally implemented on January 1. The latest "smoking control order" has expanded the scope of application, strictly controlled the scope of smoking places, and put forward the requirements of smoking ban in outdoor areas of some public places. The implementation of a multi-sectoral supervision model has strengthened law enforcement. Electronic cigarettes are also included in the smoking ban.

Chinese version

【#杭州升级控烟令#：电子烟被纳入禁烟范围，违法吸烟最高罚2万】1日，最新修订的《杭州市公共场所控制吸烟条例》正式实施。此次最新“控烟令”扩大了适用范围、严格了控制吸烟场所范围、对一些公共场所的室外区域也提出了禁烟要求。实行多部门监管模式，增强了执法力量。电子烟也被纳入禁烟范围。

RP-4

English version

Hangzhou Upgrading Smoking Control Ordinance: Electronic cigarettes were included in the smoking ban with a maximum penalty of 20,000 RMB.

The newly revised Hangzhou Regulations on Smoking Control in Public Places were formally implemented on January 1. The latest "smoking control order" has set a buffer period for smoking places. Electronic cigarettes are included in the scope of smoking ban. If operators and managers fail to fulfill their duties of smoking control, they can be fined up to 20,000 yuan.

Chinese version

【杭州升级控烟令：电子烟被纳入禁烟范围 最高罚2万】1日，最新修订的《杭州市公共场所控制吸烟条例》正式实施。此次最新“控烟令”设置了缓冲期限制吸烟场所，电子烟被纳入禁烟范围，对场所经营者、管理者未履行控烟职责的，最高可罚2万。（浙江24小时）O网页链接

RP-5

English version

Hangzhou Upgraded Smoking Control Ordinance: Electronic cigarettes were included in the smoking ban, with a maximum penalty of 20,000 RMB for illegal smoking.

The newly revised Hangzhou Regulations on Smoking Control in Public Places was formally implemented on January 1. The latest "smoking control order" has expanded the scope of application, strictly controlled the scope of smoking places, and put forward the requirements of smoking ban in outdoor areas of some public places. The implementation of a multi-sectoral supervision model has strengthened law enforcement. Electronic cigarettes are also included in the smoking ban.

Chinese version

【杭州升级控烟令：电子烟被纳入禁烟范围，违法吸烟最高罚2万】1日，最新修订的《杭州市公共场所控制吸烟条例》正式实施。此次最新“控烟令”扩大了适用范围、严格了控制吸烟场所范围、对一些公共场所的室外区域也提出了禁烟要求。实行多部门监管模式，增强了执法力量。电子烟也被纳入禁烟范围。(钱江 ) 展开全文c

RP-6

English version

Hangzhou Upgrading Smoking Control Ordinance: Electronic cigarettes were included in the smoking ban with a maximum penalty of 20,000 RMB.

The newly revised Hangzhou Regulations on Smoking Control in Public Places were formally implemented on January 1. The latest "smoking control order" has set a buffer period for smoking places. Electronic cigarettes are included in the scope of smoking ban. If operators and managers fail to fulfill their duties of smoking control, they can be fined up to 20,000 yuan.

Chinese version

【杭州升级控烟令：电子烟被纳入禁烟范围 最高罚2万】最新修订的《杭州市公共场所控制吸烟条例》正式实施。此次最新“控烟令”设置了缓冲期限制吸烟场所，电子烟被纳入禁烟范围，对场所经营者、管理者未履行控烟职责的，最高可罚2万。

RP-7

English version

# Hangzhou Hot Events #The newly revised Regulations on Smoking Control in Public Places of Hangzhou have been formally implemented. The latest "smoking control order" has expanded the scope of application, strictly controlled the scope of smoking places, and put forward the requirements of smoking ban in outdoor areas of some public places. The implementation of a multi-sectoral supervision model has strengthened law enforcement. Electronic cigarettes are also included in the smoking ban.

Chinese version

#杭州热门大事件#最新修订的《杭州市公共场所控制吸烟条例》正式实施。此次最新“控烟令”扩大了适用范围、严格了控制吸烟场所范围、对一些公共场所的室外区域也提出了禁烟要求。实行多部门监管模式，增强了执法力量。电子烟也被纳入禁烟范围。 L浙样红TV的微博视频

RP-8

English version

Hangzhou Upgrading Smoking Control Ordinance: Electronic cigarettes are included in the scope of prohibition, with a maximum penalty of 20,000 RMB for illegal smoking.

The newly revised Hangzhou Regulations on Smoking Control in Public Places have been formally implemented. The latest "smoking control order" has expanded the scope of application, strictly controlled the scope of smoking places, and put forward the requirements of smoking ban in outdoor areas of some public places. The implementation of a multi-sectoral supervision model has strengthened law enforcement. Electronic cigarettes are also included in the smoking ban.

Chinese version

【杭州升级控烟令：电子烟被纳入禁烟范围，违法吸烟最高罚2万】最新修订的《杭州市公共场所控制吸烟条例》正式实施。此次最新“控烟令”扩大了适用范围、严格了控制吸烟场所范围、对一些公共场所的室外区域也提出了禁烟要求。实行多部门监管模式，增强了执法力量。电子烟也被纳入禁烟范围。

RP-9

English version

This kind of smoke is harmful!

The newly revised Hangzhou Regulations on Smoking Control in Public Places came into effect on January 1. No smoking places not only prohibit lighting tobacco products and smoking traditional cigarettes, but also prohibit smoking electronic cigarettes. Why are electronic cigarettes banned?

Chinese version

【这种烟，害人！】1日，最新修订的《杭州市公共场所控制吸烟条例》正式实施。禁烟场所不仅禁止点燃烟草制品和吸传统卷烟，也禁止吸电子烟。电子烟为何会被明令禁止？戳图了解↓↓转给身边吸烟的TA！

RP-10

English version

# Hangzhou Headline. This kind of smoke, harmful!

The newly revised Hangzhou Regulations on Smoking Control in Public Places came into effect on January 1. No smoking places not only prohibit lighting tobacco products and smoking traditional cigarettes, but also prohibit smoking electronic cigarettes. Why are electronic cigarettes banned?

Chinese version

#杭州头条#【这种烟，害人！】1日，最新修订的《杭州市公共场所控制吸烟条例》正式实施。禁烟场所不仅禁止点燃烟草制品和吸传统卷烟，也禁止吸电子烟。电子烟为何会被明令禁止？戳图了解↓↓转给身边吸烟的TA！

RP-11

English version

Smoking is harmful to health. What about electronic cigarettes?

Let's do a science popularization.

# Hangzhou electronic cigarettes are included in the smoking ban#

Chinese version

吸烟有害健康，那电子烟呢？

我们来做个科普。

#杭州电子烟被纳入禁烟范围#

RP-12

English version

# Hangzhou electronic cigarettes are included in the smoking ban# A video tells you how harmful smoking is. After watching it, everyone decides to quit smoking. [Health preservation - Lao Yang's Weibo Video Link] @ Health preservation - LaoYang

Chinese version

#杭州电子烟被纳入禁烟范围#一个视频告诉你吸烟的危害到底有多大，看完的人都决定戒烟了！ [健康养生-老杨的微博视频链接]@健康养生-老杨

RP-13

English version

# Hangzhou electronic cigarettes are included in the smoking ban#Comprehensive tobacco control from family to society is the right way for parents to protect their children from second-hand smoke. Smoke-free and healthy environment is the best gift we give our children. [Rice cake mother 's Second Shot Video Link] @ Rice cake mother

Chinese version

#杭州电子烟被纳入禁烟范围# 从家庭到社会全面控烟，是爸妈保护孩子免受二手烟危害的正确方法。无烟健康的环境，是我们送给孩子最好的礼物。 [年糕妈妈的秒拍视频链接] @年糕妈妈
